# Mini‐consolidations or intermediate‐dose cytarabine for the post‐remission therapy of AML patients over 60. A retrospective study from the DATAML and SAL registries

**DOI:** 10.1002/ajh.27510

**Published:** 2024-11-18

**Authors:** Christian Récher, Pierre‐Yves Dumas, Emilie Bérard, Suzanne Tavitian, Thibaut Leguay, Jean Galtier, Camille Alric, Audrey Bidet, Eric Delabesse, Jean Baptiste Rieu, Jean‐Philippe Vial, François Vergez, Isabelle Luquet, Emilie Klein, Anne‐Charlotte de Grande, Audrey Sarry, Sven Zukunft, Uwe Platzbecker, Carsten Müller‐Tidow, Claudia D. Baldus, Martin Bornhäuser, Hubert Serve, Sarah Bertoli, Arnaud Pigneux, Christoph Röllig

**Affiliations:** ^1^ Institut Universitaire du Cancer de Toulouse Oncopole, Centre Hospitalier Universitaire de Toulouse Université Toulouse III Paul Sabatier Toulouse France; ^2^ Institut National de la Santé et de la Recherche Médicale, U1312, Service d'Hématologie Clinique et de Thérapie Cellulaire Université de Bordeaux, CHU Bordeaux Bordeaux France; ^3^ Service d'Epidémiologie, CERPOP, Inserm, Centre Hospitalier Universitaire de Toulouse Université Toulouse III Paul Sabatier Toulouse France; ^4^ Laboratoire d'Hématologie Biologique CHU Bordeaux Bordeaux France; ^5^ Laboratoire d'Hématologie Biologique, Institut Universitaire du Cancer de Toulouse Oncopole Centre Hospitalier Universitaire de Toulouse Toulouse France; ^6^ Medizinische Klinik und Poliklinik I Universitätsklinikum TU Dresden Dresden Germany; ^7^ Klinik und Poliklinik für Hämatologie, Zelltherapie Hämostaseologie und Infektiologie Universitätsklinikum Leipzig Leipzig Germany; ^8^ Klinik für Hämatologie, Onkologie und Rheumatologie Universitätsklinikum Heidelberg Heidelberg Germany; ^9^ Klinik für Innere Medizin II Universitätsklinikum Schleswig‐Holstein Kiel Germany; ^10^ Medizinische Klinik 2 Universitätsklinikum Frankfurt Frankfurt/Main Germany

## Abstract

According to current recommendations, older AML patients in first complete remission (CR) after induction chemotherapy should receive consolidation with intermediate‐dose cytarabine (IDAC). However, no study has demonstrated the superiority of IDAC over other regimen. In this retrospective study, we compared the efficacy of mini‐consolidations (idarubicin 8 mg/m^2^ day 1, cytarabine 50 mg/m^2^/12 h, day 1–5) and IDAC. Inclusion criteria were newly diagnosed AML, age > 60 years, first CR after induction and at least 1 cycle of consolidation. Of the 796 included patients, 322 patients received mini‐consolidations and 474 patients received IDAC. Mini‐consolidation patients were older, and more often, they had de novo AML and unfavorable risk. The rate of allogeneic transplantation was higher in the IDAC group. The median number of cycles was higher in the mini‐consolidation group (4 vs. 2; *p* < .0001). Median relapse‐free survival was 18 months with mini‐consolidations and 12 months with IDAC (*p* = .0064). In multivariate analysis, the risk of relapse or death was significantly higher in the IDAC group (*p* = .004). Median OS was 36 versus 31 months with mini‐consolidations or IDAC, respectively (*p* = .46). In multivariate analysis, the consolidation regimen had no significant influence on OS (*p* = .43). In older AML patients, post‐remission therapy with mini‐consolidations represents an alternative to IDAC.

## 1 INTRODUCTION

In older patients with newly diagnosed acute myeloid leukemia (AML) who are suitable for intensive treatment, induction chemotherapy remains the established therapeutic option associated with long‐term survival.^1^, ^2^ However, for 60%–70% of those older patients who achieve complete remission (CR), the optimal post‐remission consolidation therapy has not been clearly defined by prospective clinical trials and remains a matter of debate.^3^, ^4^ In the seminal study of the Cancer and Leukemia Group B (CALGB) that established high dose of cytarabine (HDAC) as a standard for post‐remission therapy in patients younger than 60 years of age, there was no difference in relapse‐free survival (RFS) and overall survival (OS) in patients >60 years randomized to receive four courses of cytarabine at 100 mg/m^2^/day, 400 mg/m^2^/day (continuous infusion over 5 days), or 3 g/m^2^ (over a 3 h infusion each 12 h on days 1, 3, and 5).^5^ The CALGB group also compared standard‐dose cytarabine (SDAC) at 100 mg/m^2^ every 12 h for 5 days with an intensified schedule of cytarabine 500 mg/m^2^ every 12 h associated with mitoxantrone for 3 days in older patients without any difference in RFS and OS.^6^ The Acute Leukemia French Association (ALFA) 9803 clinical trial comparing a single “3 + 7” consolidation course with an outpatient schedule consisting of six cycles of single‐dose anthracycline combined with subcutaneous cytarabine at 60 mg/m^2^ every 12 h for 5 days in AML patients over 65 years showed an OS advantage in favor of the repeated courses of mini‐consolidations.^7^ Both trials also demonstrated lower toxicity with reduced‐intensity regimens. In line with these studies, the 2017 ELN guidelines indicated that there was no established value of intensive consolidation therapy in older patients with intermediate or adverse‐risk genetics.^8^ The 2022 ELN recommendations, however, propose that patients ineligible for allogeneic hematopoietic stem cell transplantation (allo‐HSCT) should receive consolidation therapy with intermediate‐dose cytarabine (IDAC, 500–1000 mg/m^2^/12 h, d1‐3, for 3–4 cycles), mainly because this schedule has become popular in practice, as it could mimic the cytarabine dose effect observed in younger patients without too much toxicity.

The DATAML centers traditionally use the consolidation schema of the French Innovative Leukemia Organization (FILO) study group including up to six outpatient mini‐consolidation cycles for most patients although some patients may occasionally receive IDAC.^9^, ^10^ In a recent retrospective study, we compared both strategies in patients of the DATAML registry and showed no difference in RFS and OS.^11^

The aim of this study was to compare the efficacy and tolerability of the mini‐consolidations used in the French DATAML registry and IDAC used routinely by the German Study Alliance Leukemia (SAL) group in a larger series of AML patients >60 years in first complete remission after intensive induction chemotherapy. The primary objective was to compare OS in patients treated with mini‐consolidations or IDAC. Secondary objectives were to compare RFS and cumulative incidence of relapse (CIR).

## 2 SUBJECTS AND METHODS

### 2.1 Patients

The inclusion criteria for this retrospective study were newly diagnosed AML between January 1, 2010, and December 31, 2019, age > 60 years, first CR after one or two “3 + 7” induction cycles, consolidation treatment with at least 1 cycle of mini‐consolidation or IDAC. Patients with acute promyelocytic leukemia or AML with *t*(9;22)/*BCR::ABL1*, patients treated with CPX‐351 or patients who had received both types of consolidation (mini‐consolidation and IDAC) were not included. A minimal data set extracted from DATAML and SAL registries included the variables age, sex, ECOG (eastern cooperative oncology group) performance status at diagnosis, history of cytotoxic treatment, date of diagnosis, AML status (de novo or secondary), white blood cell count, 2017 ELN risk classification, mutational status, nature of first‐line therapy, allo‐HSCT in first complete remission or after relapse, date of day 1 of first consolidation cycle, number of post‐remission cycles, and date of relapse and/or death. This study was performed in accordance with the Declaration of Helsinki. All registries were approved by institutional review boards or national authorities, and informed consent was obtained from all patients.

### 2.2 Treatments and endpoints

Mini‐consolidation treatment included cytarabine 50 mg/m^2^/12 h on days 1–5 by subcutaneous injection and idarubicin 8 mg/m^2^ on day 1 performed on an outpatient basis (up to 6 cycles every 30 to 45 days). IDAC was cytarabine 1–1.5 g/m^2^/12 h on days 1–3 or 1, 3, and 5 (up to 3 cycles). Allo‐HSCT was proposed in eligible patients with intermediate‐risk AML or adverse‐risk AML according to local practices. CIR, RFS, and OS were defined according to the European LeukemiaNet (ELN) criteria.^1^

### 2.3 Statistical analysis

Patients characteristics were described using numbers and frequencies for qualitative data, and median, interquartile range (IQR), and range (minimum−maximum) for quantitative data. Categorical variables were compared between IDAC and mini‐consolidations arms using the chi‐squared test (or Fisher's exact test when necessary). Student's *t* test was used to compare the distributions of continuous data (Mann‐Whitney's test was used when the distribution departed significantly from normality or when homoscedasticity was rejected). For OS and RFS, differences in survival functions between the IDAC and mini‐consolidations arms were described using survival at 1, 3, and 5 years together with median with IQR and were tested using the log‐rank test. For relapse (CIR), cumulative incidence functions were drawn (as non‐relapse mortality was used as a competing event) and compared using Gray's test. Adjusted hazard ratios (HR) and 95% confidence intervals (95%CI) were assessed using a standard Cox model for OS and RFS and a proportional sub‐distribution hazard model (an extension of the Cox model) for competing risks for CIR.^12^ Multivariate analyses included IDAC versus mini‐consolidations arms together with potential confounding factors (age, gender, performance status, WBC, 2017 ELN risk, secondary vs. de novo AML, *NPM1* and *FLT3*‐ITD mutations, study period, and allo‐HSCT in first complete remission) associated with the endpoints (to the threshold of *p* < .20). Stepwise regression analysis was then used to assess variables that were significantly and independently associated with the endpoints (*p* < .05). The proportional hazard assumption was tested for each covariate of the Cox model using log–log plot curves and was always supported. When the linear hypothesis was not supported, continuous potential confounding factors were transformed into ordered data. Interactions between all potential confounding factors and IDAC versus mini‐consolidations were tested. None were significant, indicating that the effect of treatment (IDAC vs. mini‐consolidations) was not significantly different according to all confounding factors analyzed, especially according to age, 2017 ELN risk, *NPM1* mutation, or allo‐HSCT in first CR. Allo‐HSCT in first CR was evaluated as a time‐dependent qualitative covariate. To better appreciate the impact of IDAC versus mini‐consolidations, we used the propensity score method to more extensively take into account potential baseline differences between IDAC versus mini‐consolidations arms. A multivariate logistic regression model was generated to estimate for each patient a propensity score to receive IDAC versus mini‐consolidations. Covariates were all variables expected to be associated with IDAC versus mini‐consolidations in clinical practice (age, gender, performance status, WBC, 2017 ELN risk, secondary vs. de novo AML, *NPM1* and *FLT3*‐ITD mutations, study period, history of cytotoxic treatment, and targeted therapy added to induction and inclusion in a clinical trial). The performance of the model was estimated with the Hosmer–Lemeshow chi‐squared statistic and the C‐statistic. Based on propensity score, subjects with IDAC were matched with patients with mini‐consolidations. In the subgroup of propensity score matched patients, endpoints were compared between IDAC and mini‐consolidations arms, after adjustment for allo‐HSCT (as time‐dependent variable). All reported *p*‐values were two‐sided, and the significance threshold was <.05. Statistical analyses were performed using STATA® version 18.0 (STATA Corp., College Station, TX).

## 3 RESULTS

### 3.1 Study population

This retrospective study included 796 patients >60 years who had obtained a first CR after intensive induction chemotherapy between January 1, 2010, and December 31, 2019. Post‐remission therapy was mini‐consolidations in 322 patients (40.5%) from the DATAML registry and IDAC in 474 patients (59.5%) from the SAL registry. Their characteristics are presented in Table 1. Compared to patients treated with IDAC, patients treated with mini‐consolidation were slightly older (47.5% > 70 years vs. 40.1%; *p* = .037), and more often, they had de novo AML (81.4% vs. 75.3%; *p* = .044) and adverse risk according to 2017 ELN classification (22% vs. 12%; *p <* .001). There were no differences in terms of sex, performance status at diagnosis, white blood cell (WBC) count, *FLT3*‐ITD, or *NPM1* mutations. Induction therapy consisted mainly of idarubicin–cytarabine–lomustine (76.4%) in the mini‐consolidation group and daunorubicin–cytarabine (96%) in the IDAC group. Midostaurin was added to induction chemotherapy in 14 patients (4.3%) of the mini‐consolidation group and 3 patients (0.6%) of the IDAC group (*p* < .001). There were 94 patients (29.2%) of the mini‐consolidation group and 59 patients (12.4%) of the IDAC group (*p* < .001) included in a clinical trial at induction. The median number of consolidation cycles was 4 (IQR, 2–6) with mini‐consolidations and 2 (IQR, 1–3) with IDAC (*p* < .0001). The rate of allo‐HSCT was higher in the IDAC group both in first CR (17.5% vs. 11.5%, *p* = .019) and after relapse (30.5% vs. 9.5%, *p* < .0001).

**TABLE 1:** Characteristics of patients according to post‐remission therapy.

	Mini‐consolidations	IDAC	*p*‐value	Total
	322 (40.5%)	474 (59.5%)		796 (100.0%)
Sex—*n* (%)				
Male	178 (55.3)	256 (54.0)	.723	434 (54.5)
Female	144 (44.7)	218 (46.0)		362 (45.5)
Study period—*n* (%)				
2010–2014	139 (43.2)	260 (54.9)	.001	399 (50.1)
2015–2019	183 (56.8)	214 (45.1)		397 (49.9)
Age (years)				
Median	69.4	69.0	.015	69.00
Min–Max	61.1; 82.8	61.0; 86.0		61.0; 86.0
Age (years)—*n* (%)				
≤70	169 (52.5)	284 (59.9)	.037	453 (56.9)
>70	153 (47.5)	190 (40.1)		343 (43.1)
AML status—*n* (%)				
De novo	262 (81.4)	354 (75.3)	.044	616 (77.8)
Secondary	60 (18.6)	116 (24.7)		176 (22.2)
History of cytotoxic treatment—*n* (%)				
None	283 (87.9)	435 (91.8)	<.0001	718 (90.2)
Chemotherapy	6 (1.9)	22 (4.6)		28 (3.5)
Radiotherapy	18 (5.6)	11 (2.3)		29 (3.6)
Chemotherapy + radiotherapy	7 (2.2)	6 (1.3)		13 (1.6)
Others	8 (2.5)	0 (0.0)		8 (1.0)
Performance status—*n* (%)				
0–1	262 (83.2)	381 (82.1)	.701	643 (82.5)
2–3–4	53 (16.8)	83 (17.9)		136 (17.5)
WBC (Giga/L)				
*n*/missing	321/1	472/2	.466	793/3
Median	5.2	7.4		6.2
IQR	2.1; 28.1	2.0;41.8		2.1;35.3
Min–Max	0.4;359.4	0.2;305.0		0.2;359.4
WBC (Giga/L)—*n* (%)				
<30	243 (75.7)	332 (70.3)	.096	575 (72.5)
≥30	78 (24.3)	140 (29.7)		218 (27.5)
2017 ELN risk—*n* (%)				
Favorable	98 (39.2)	152 (34.8)	<.001	250 (36.4)
Intermediate	96 (38.4)	231 (52.9)		327 (47.6)
Adverse	56 (22.4)	54 (12.4)		110 (16.0)
*FLT3*‐ITD—*n* (%)				
No	223 (80.5)	334 (80.7)	.955	557 (80.6)
Yes	54 (19.5)	80 (19.3)		134 (19.4)
*NPM1* mutation—*n* (%)				
No	161 (58.1)	262 (59.8)	.653	423 (59.2)
Yes	116 (41.9)	176 (40.2)		292 (40.8)
Induction chemotherapy—*n* (%)				
Daunorubicin–cytarabine	6 (1.9)	455 (96.0)	<.0001	461 (57.9)
Idarubicin–cytarabine	65 (20.2)	1 (0.2)		66 (8.3)
Idarubicin–cytarabine–lomustine	246 (76.4)	0 (0.0)		246 (30.9)
Daunorubicin–cytarabine–GO	0 (0.0)	2 (0.4)		2 (0.3)
Other	5 (1.6)	16 (3.4)		21 (2.6)
Clinical trial—*n* (%)				
No	228 (70.8)	415 (87.6)	<.0001	643 (80.8)
Yes	94 (29.2)	59 (12.4)		153 (19.2)
Allo‐HSCT				
In first CR—*n* (%)				
No	285 (88.5)	391 (82.5)	.019	676 (84.9)
Yes	37 (11.5)	83 (17.5)		120 (15.1)
After relapse—*n* (%)				
No	191 (90.5)	210 (69.5)	<.0001	401 (78.2)
Yes	20 (9.5)	92 (30.5)		112 (21.8)

Abbreviations: Allo‐HSCT, allogeneic hematopoietic stem cell transplantation; ELN, European LeukemiaNet; GO, gemtuzumab ozogamycin; IQR, interquartile range; Max, maximum; Min, minimum; WBC, white blood cell count.

### 3.2 Overall survival

**FIGURE 1:**
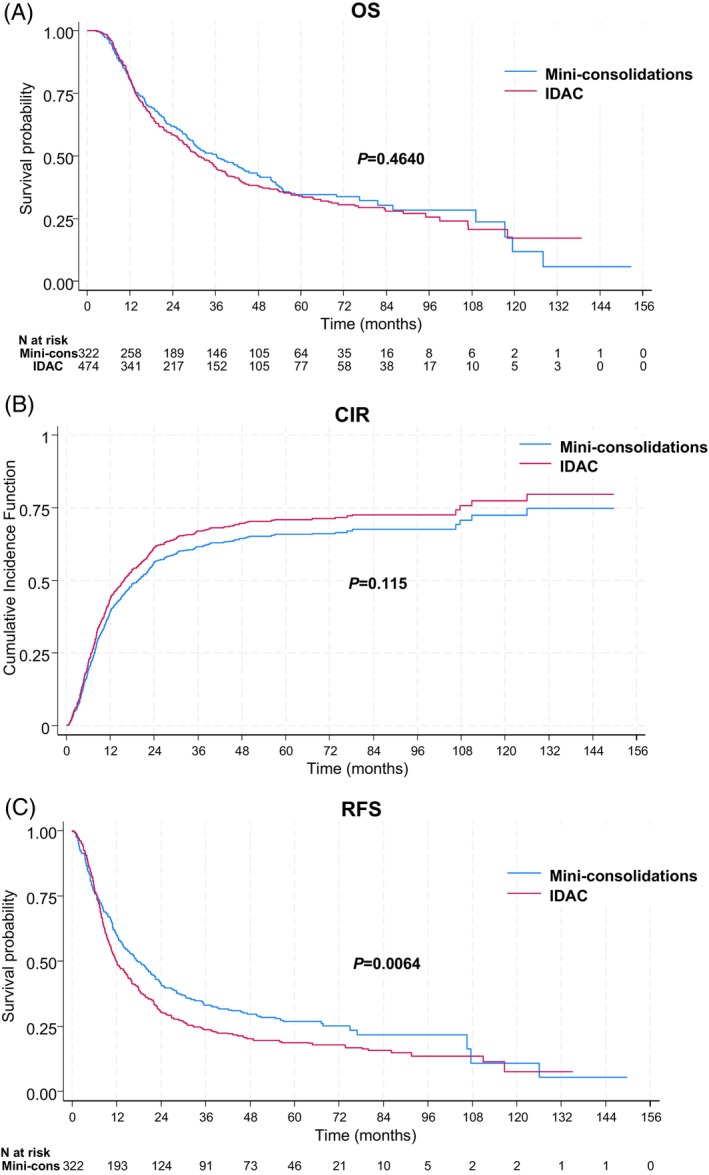
Kaplan–Meier curves for overall survival (A), cumulative incidence of relapse (B), and relapse‐free survival (C). [Color figure can be viewed at wileyonlinelibrary.com]

The median follow‐up was 61 months (interquartile range, IQR, 40–81) for the whole population, 63 (IQR, 50–74) in the mini‐consolidation group and 58 months (IQR, 28–87) in the IDAC group. Median OS was 36 (IQR, 14–109) and 31 months (IQR, 14–99) in the mini‐consolidation and IDAC groups, respectively (*p* = .46) (Figure 1A). OS at 1, 3, and 5 years was 80% (95% confidence interval [CI]: 75–84) vs. 81% (95% CI: 77–84), 51 (95% CI: 45–56) vs. 46% (95% CI: 41–51), and 35% (95% CI: 29–40) vs. 34% (95% CI: 29–39) in the mini‐consolidation and IDAC groups, respectively. Univariate and multivariate analyses are shown in Table 2. In the multivariate analysis, consolidation type was not significantly and independently associated with OS (*p* = .425). Adjustment for the number of consolidation cycles (<2 vs. ≥2) did not modify the impact of consolidation type (*p* = .575).

**TABLE 2:** Univariate and multivariate analyses for overall survival.

	Number	Events	Univariate			Multivariate		
			HR	95% CI	*p*‐value	HR	95% CI	*p*‐value
Treatment								
Mini‐consolidations	322	203	1			1		
IDAC	474	269	1.07	0.89;1.29	.464	1.08	0.89;1.31	.425
Study period								
2010–2014	399	272	1					
2015–2019	397	200	0.82	0.69;0.99	.041			
Sex								
Male	434	261	1					
Female	362	211	1.00	0.83;1.19	.969			
Age (years)								
≤70	453	251	1			1		
>70	343	221	1.31	1.09;1.57	.004	1.35	1.12;1.62	.002
AML status								
De novo	616	353	1					
Secondary	176	115	1.17	0.95;1.45	.142			
Performance status at diagnosis								
0–1	643	374	1			1		
2–3–4	136	88	1.37	1.09;1.73	.008	1.35	1.06;1.71	.014
WBC (Giga/L)								
<30	575	328	1			1		
≥30	218	141	1.27	1.04;1.55	.017	1.22	1.00;1.50	.050
2017 ELN risk								
Favorable	250	127	1			1		
Intermediate	327	207	1.33	1.07;1.66	.011	1.39	1.11;1.74	.004
Adverse	110	72	1.74	1.30;2.33	<.001	1.79	1.34;2.40	<.001
*FLT3*‐ITD								
No	557	328	1					
Yes	134	83	1.14	0.90;1.45	.283			
*NPM1* mutation								
No	423	262	1					
Yes	292	157	0.81	0.66;0.99	.036			
Allo‐HSCT								
No	676	410	1					
Yes	120	62	0.82	0.62;1.07	.139			

Abbreviations: Allo‐HSCT, allogeneic hematopoietic stem cell transplantation; CI, confidence interval; ELN, European LeukemiaNet; HR, hazard ratio; WBC, white blood cell count.

### 3.3 Cumulative incidence of relapse and relapse‐free survival

In the mini‐consolidation group, 209 patients relapsed and the CIR was 39%, 56%, and 62% at 1, 2, and 3 years, respectively. In the IDAC group, 302 patients relapsed and the CIR was 44%, 61%, and 67% at 1, 2, and 3 years, respectively (Figure 1B). Univariate and multivariate analyses for CIR are shown in Table S1. In multivariate analysis, the relapse risk was significantly and independently higher in patients treated with IDAC (HR 1.30, 95% CI: 1.1–1.6; *p* = .006). Adjustment for the number of consolidation cycles did not modify the impact of IDAC on the relapse risk (HR 1.28, 95% CI: 1.1–1.5; *p* = .012).

Median RFS was 18 (IQR, 7–75) and 12 months (IQR, 7–33) in the mini‐consolidation and IDAC groups, respectively (*p* = .006) (Figure 1C). RFS at 1, 3, and 5 years was 60% (95% CI: 54–65) versus 50% (95% CI: 45–54), 33% (95% CI: 28–38) versus 24% (95% CI: 20–28), and 27% (95% CI: 22–32) versus 19% (95% CI: 15–23) in the mini‐consolidation and IDAC groups, respectively. Univariate and multivariate analyses are shown in Table 3. In multivariate analysis, the risk of relapse or death was significantly and independently higher in patients treated with IDAC (HR 1.29, 95% CI: 1.1–1.5; *p* = .004). Adjustment for the number of consolidation cycles did not modify the impact of IDAC on RFS (HR 1.26, 95% CI: 1.1–1.5; *p* = .011).

**TABLE 3:** Univariate and multivariate analyses for relapse‐free survival.

	Number	Events	Univariate			Multivariate		
			HR	95% CI	*p*‐value	HR	95% CI	*p*‐value
Treatment								
Mini‐consolidations	322	233	1			1		
IDAC	474	349	1.26	1.07;1.49	.007	1.29	1.09;1.54	.004
Study period								
2010–2014	399	314	1					
2015–2019	397	265	0.87	0.74;1.02	.089			
Sex								
Male	434	319	1					
Female	362	263	0.98	0.83;1.15	.803			
Age (years)								
≤70	453	317	1					
>70	343	265	1.21	1.03;1.42	.024			
AML status								
De novo	616	445	1					
Secondary	176	133	1.15	0.94;1.39	.170			
Performance status at diagnosis								
0–1	643	467	1					
2–3–4	136	102	1.22	0.99;1.52	.066			
WBC (Giga/L)								
<30	575	411	1			1		
≥30	218	168	1.18	0.99;1.41	.072	1.24	1.03;1.50	0.026
2017 ELN risk								
Favorable	250	171	1					
Intermediate	327	247	1.16	0.95;1.41	.145	^a^		
Adverse	110	84	1.40	1.08;1.82	.012	1.31	1.02;1.68	0.032
*FLT3*‐ITD								
No	557	413	1					
Yes	134	99	1.09	0.88;1.36	.435			
*NPM1* mutation								
No	423	325	1			1		
Yes	292	200	0.82	0.68;0.97	.024	0.75	0.62;0.91	0.004
Allo‐HSCT								
No	676	510	1			1		
Yes	120	72	0.68	0.53;0.87	.003	0.59	0.46;0.76	<0.001

Abbreviations: Allo‐HSCT, allogeneic hematopoietic stem cell transplantation; CI, confidence interval; ELN, European LeukemiaNet; HR, hazard ratio; WBC, white blood cell count.

^a^ Adverse versus favorable/intermediate (=1).

### 3.4 Propensity score matching

To better appreciate the impact of mini‐consolidations versus IDAC on OS, CIR, and RFS, we used the propensity score method to take into account potential baseline differences between patients treated with mini‐consolidations in the DATAML registry and those treated with IDAC in the SAL registry. A multivariate logistic regression model was generated to estimate for each patient a propensity score to receive mini‐consolidations or IDAC. Covariates were all variables expected to be associated with mini‐consolidations versus IDAC (age, gender, performance status, WBC, 2017 ELN risk, secondary versus de novo AML, *NPM1* and *FLT3*‐ITD mutations, study period, history of cytotoxic treatment, and targeted therapy added to induction and inclusion in a clinical trial). The performance of the model was appreciated with the Hosmer–Lemeshow chi‐squared statistic (*p*‐value = .456) and the c‐statistic (0.73, 95% CI: 0.69–0.76). Mean propensity score was 0.503 (±0.209) in the mini‐consolidation group (*n* = 322) and 0.341 (±0.162) in the IDAC group (*n* = 470). According to the propensity score, 214 subjects treated with mini‐consolidations were matched with 214 subjects with IDAC (206 with a precision of 0.0001, 6 with a precision of 0.001, 64 with a precision of 0.01, and 152 with a precision of 0.1). Mean propensity score was the same in both groups (0.407 ± 0.161) in the matched sample. OS, CIR, and RFS were compared between mini‐consolidation and IDAC treatment in the subgroup of propensity score matched patients after adjustment for allo‐HSCT (as time‐dependent variable). The results remained unchanged compared with the findings of the multivariate analyses in the whole patient population (Table S2).

## 4 DISCUSSION

This study comparing two therapeutic strategies for post‐remission therapy showed that mini‐consolidations including a single anthracycline dose and standard dose of cytarabine represent an alternative to the recommended IDAC regimen in AML patients older than 60 years who are in first CR after induction chemotherapy. Of note, no significant interaction between treatment (mini‐consolidations vs. IDAC) and classical confounding factors was found, indicating that the effect of consolidation regimen was not different in subgroups and in particular according to age, 2017 ELN risk, *NPM1* mutation, or allo‐HSCT (analyzed as a time‐dependent variable).

Although overall survival was similar with both strategies, the post‐remission therapy with mini‐consolidations was associated with a lower incidence of relapses and a better relapse‐free survival. The explanations for this difference are unclear. Despite adjustment for the number of cycles, it is possible that the higher number of treatment cycles in the mini‐consolidation group contributed to this result by increasing the duration of exposure to genotoxic treatment. A similar finding was observed in the randomized trial of the ALFA group comparing 6 cycles of mini‐consolidations and a single intensive chemotherapy cycle in patients older than 65 years.^7^ Alternatively, the synergistic combination of standard‐dose cytarabine and idarubicin could have induced a stronger anti‐leukemic activity on residual disease. In younger AML patients, multidrug regimens combining anthracyclines and IDAC or HDAC have shown similar or even greater efficacy than IDAC/HDAC, notably in high‐risk patients, but with increased toxicity.^13^, ^14^, ^15^ Lastly, differences in induction chemotherapy may also have played a role. Lomustine used in patients of the DATAML registry has been associated with better outcome in older AML patients while comparisons between idarubicin and daunorubicin yielded heterogeneous results.^9^, ^16^, ^17^, ^18^ However, effective salvage options and equally distributed genetically determined disease biology in both treatment groups led to similar overall survival in the long term.

Demonstrating a similar outcome of mini‐consolidations over IDAC in terms of OS may have important implications. Indeed, two recent studies by the DATAML registry in France showed that mini‐consolidations were associated with a considerable reduction in infection rates, pancytopenia duration, transfusion requirements, and hospitalization stays compared to IDAC. Patients receiving mini‐consolidations spent on average 20 days less in hospital over the whole period of post‐remission treatment.^11^ Importantly, an economic analysis, which performed from the French National Health Insurance perspective and focused on costs associated with inpatient stays, showed that the mini‐consolidation strategy could save up to 30 000 euros per patient compared to IDAC in the French health care system.^19^ Comparing the two approaches, we can conclude that patients can be treated on an outpatient basis, are longer on treatment, and increase their cumulative anthracyclin dose with mini‐consolidation. On the other hand, total treatment time with IDAC is shorter, associated with more pronounced cytopenia, and usually requiring inpatient treatment.

The limitations of this study include its retrospective and non‐randomized nature. There was heterogeneity in induction chemotherapy regimen, and we also recognize that the number of six mini‐consolidation cycles has not been established by clinical trials but rather by clinical practice, and therefore the optimal number of mini‐consolidation cycles is yet to be defined. Moreover, maintenance therapy with oral azacitidine was not approved during the study period and we can only speculate that this treatment might have limited the risk of relapse after IDAC in our study.^20^ Nevertheless, the multivariate analysis based on a large cohort of patients (*n* = 796) and the propensity score enables us to draw reliable conclusions on the absence of difference between the two strategies in terms of OS.

Consolidation treatment of older AML patients remains an evolving field with the advent of new drugs and the widespread use of allo‐HSCT and maintenance. It should deepen the response to induction therapy without triggering major toxicity. The OS results of our study show the curative potential of current state‐of‐the‐art chemotherapy and at the same time demonstrate the need for further improvement. In this respect, it may be worth exploring whether either or both consolidation approaches may be suited for the addition of new drugs such as venetoclax or quizartinib without excessive hematological toxicity and/or the replacement of anthracyclines to further increase the curative potential and proportion of long‐term survivors diagnosed with AML.

## AUTHOR CONTRIBUTIONS

CR, PYD, EB, ST, TL, JG, CA, AB, ED, JBR, JPV, FV, IL, EK, ACdG, AS, SZ, UP, CMT, CB, MB, HS, SB, AP, and CRö collected data, treated patients, or performed biological analyses. EB performed statistical analysis and wrote the paper. CR collected data, treated patients, supervised analysis, and wrote the paper. All authors reviewed, provided comments, and approved the manuscript.

## CONFLICT OF INTEREST STATEMENT

Christian Récher declares a consulting or advisory role with AbbVie, Amgen, Astellas, BMS, Boehringer, Jazz Pharmaceuticals, J&J, and Servier, research funding from AbbVie, Amgen, Astellas, BMS, Iqvia, and Jazz Pharmaceuticals, and support for attending meetings and/or travel from AbbVie, Novartis, and Servier. Pierre‐Yves Dumas declares a consulting or advisory role for Daiichi‐Sankyo, Astellas, Novartis, AbbVie, Servier, BMS, Jazz Pharmaceutical, and Janssen, research funding (to institution) from Daiichi‐Sankyo, Astellas, Novartis, Servier, BMS, Roche, and Iqvia, and support for attending meetings and/or travel from AbbVie, Gilead, and Lilly.François Vergez declares research grants from Pierre Fabre and Roche, advisor for Astellas and Amgen. Uwe Platzbecker declares Honoraria and Research support from BMS, AbbVie, Curis, Jazz, and Ryvu. Isabelle Luquet declares an advisory role for Jazz Pharmaceuticals. Carsten Müller‐Tidow declares institutional research funding from Pfizer and BiolineRx. LR declares institutional research funding from AbbVie and honoraria from BeiGene, Jazz, and Neovii. Claudia Baldus declares advisory honoraria from Astellas, Bristol‐Meyer‐Squibb, Jazz, Janssen, Pfizer, Servier, Amgen, and Astra Zeneca. Martin Bornhäuser declares honoraria from Jazz Pharmaceuticals Advisory Board: ActiTrexx; employment by University Hospital TU Dresden, King's College London. Sarah Bertoli declares a consulting or advisory role with AbbVie, Astellas, BMS‐Celgene, Jazz Pharmaceuticals, and Servier and travel grants from AbbVie and Pfizer. Arnaud Pigneux declares a consulting or advisory role with Astellas, BMS, Servier, AbbVie, Gilead, Jazz Pharmaceuticals, Novartis, and Pfizer, research funding from Astellas, BMS, Roche, and Servier, and support for attending meetings and/or travel from Servier and AbbVie. Christoph Röllig declares institutional research funding by AbbVie, Novartis, and Pfizer and advisory honoraria from AbbVie, Astellas, Bristol‐Meyer‐Squibb, Jazz, Janssen, Novartis, Otsuka, Pfizer, and Servier. All other authors declare no competing interests.

## PATIENT CONSENT STATEMENT

Informed consent was obtained from all patients.

## Supporting information


Table S1–S2

## Data Availability

The data sets generated during and/or analyzed during the current study are available from the corresponding author upon reasonable request.
