# Supplementary material for: Mini‐consolidations or intermediate‐dose cytarabine for the post‐remission therapy of AML patients over 60. A retrospective study from the DATAML and SAL registries
Source: Am J Hematol. 2024 Nov 18;100(1):23–32. doi: 10.1002/ajh.27510 (PMC11625969; doi:10.1002/ajh.27510)
Supplement: Supplementary file 1 — Table S1–S2 [file AJH-100-23-s001.docx]

**Supplementary Table 1: Univariate and multivariate analysis for cumulative incidence of relapse**

|  |  |  | **Univariate** | | | **Multivariate** | | |
| --- | --- | --- | --- | --- | --- | --- | --- | --- |
|  | **Number** | **Events** | **HR** | **95% CI** | ***P*-value** | **HR** | **95% CI** | ***P*-value** |
| **Treatment**  Mini-consolidations  IDAC | 322  474 | 209  302 | 1  1.15 | 0.97;1.37 | 0.115 | 1  1.30 | 1.08;1.56 | 0.006 |
| **Study period**  2010-2014  2015-2019 | 399  397 | 270  241 | 1  0.90 | 0.76;1.07 | 0.224 |  |  |  |
| **Sex**  Male  Female | 434  362 | 279  232 | 1  1.00 | 0.84;1.19 | 0.973 |  |  |  |
| **Age (years)**  ≤70  >70 | 453  343 | 264  247 | 1  1.42 | 1.20;1.69 | <0.001 | 1  1.22 | 1.02;1.46 | 0.031 |
| **AML status**  De novo  Secondary | 616  176 | 394  113 | 1  1.05 | 0.85;1.30 | 0.630 |  |  |  |
| **Performance status at diagnosis**  0-1  2-3-4 | 643  136 | 408  92 | 1  1.28 | 1.01;1.64 | 0.045 |  |  |  |
| **WBC (G/L)**  <30  ≥30 | 575  218 | 365  144 | 1  1.12 | 0.92;1.38 | 0.264 |  |  |  |
| **2017 ELN risk**  Favorable  Intermediate  Adverse | 250  327  110 | 155  206  78 | 1  0.99  1.35 | 0.81;1.22  1.02;1.80 | 0.936  0.035 | *  1.58 | 1.23;2.02 | <0.001 |
| ***FLT3*-ITD**  No  Yes | 557  134 | 356  92 | 1  1.23 | 0.96;1.56 | 0.100 |  |  |  |
| ***NPM1* mutation**  No  Yes | 423  292 | 280  178 | 1  0.90 | 0.74;1.09 | 0.266 |  |  |  |
| **Allo-HSCT**  No  Yes | 676  120 | 472  39 | 1  0.35 | 0.25;0.49 | <0.001 | 1  0.34 | 0.24;0.49 | <0.001 |

HR, hazard ratio. CI, confidence interval; WBC, white blood cell count; ELN, European LeukemiaNet; Allo-HSCT, allogeneic hematopoietic stem cell transplantation.

* adverse *vs.* favorable/intermediate (=1).

**Supplementary Table 2: Multivariate analysis for OS, RFS and CIR in the propensity score matched patients.**

|  | **Number** | **Events** | **HR** | **95% CI** | ***P*-value** |
| --- | --- | --- | --- | --- | --- |
|  |  | ***Overall survival*** | | | |
| **Treatment**  Mini-consolidations  IDAC | 214  214 | 137  117 | 1  1.06 | 0.83;1.36 | 0.643 |
| **Allo-HSCT**  No  Yes | 362  66 | 223  31 | 1  0.68 | 0.46;0.99 | 0.044 |
|  |  | ***Relapse free survival*** | | | |
| **Treatment**  Mini-consolidations  IDAC | 214  214 | 159  154 | 1  1.25 | 1.00;1.57 | 0.049 |
| **Allo-HSCT**  No  Yes | 362  66 | 276  37 | 1  0.58 | 0.41;0.82 | 0.002 |
|  |  | ***Cumulative incidence of relapse*** | | | |
| **Treatment**  Mini-consolidations  IDAC | 214  214 | 139  137 | 1  1.32 | 1.04;1.67 | 0.021 |
| **Allo-HSCT**  No  Yes | 362  66 | 256  20 | 1  0.30 | 0.18;0.49 | <0.001 |

HR, hazard ratio; CI, confidence interval; Allo-HSCT, allogeneic hematopoietic stem cell transplantation.
